# Term sets: A transparent and reproducible representation of clinical code sets

**DOI:** 10.1371/journal.pone.0212291

**Published:** 2019-02-14

**Authors:** Richard Williams, Benjamin Brown, Evan Kontopantelis, Tjeerd van Staa, Niels Peek

**Affiliations:** 1 Greater Manchester Patient Safety Translational Research Centre, University of Manchester, Manchester, United Kingdom; 2 Division of Informatics, Imaging and Data Science, The University of Manchester, Manchester, United Kingdom; 3 Centre for Primary Care, Division of Population Health, Health Services Research and Primary Care, The University of Manchester, Manchester, United Kingdom; Liverpool John Moores University, UNITED KINGDOM

## Abstract

**Objective:**

Clinical code sets are vital to research using routinely-collected electronic healthcare data. Existing code set engineering methods pose significant limitations when considering reproducible research. To improve the transparency and reusability of research, these code sets must abide by FAIR principles; this is not currently happening. We propose ‘term sets’, an equivalent alternative to code sets that are findable, accessible, interoperable and reusable.

**Materials and methods:**

We describe a new code set representation, consisting of natural language inclusion and exclusion terms (term sets), and explain its relationship to code sets. We formally prove that any code set has a corresponding term set. We demonstrate utility by searching for recently published code sets, representing them as term sets, and reporting on the number of inclusion and exclusion terms compared with the size of the code set.

**Results:**

Thirty-one code sets from 20 papers covering diverse disease domains were converted into term sets. The term sets were on average 74% the size of their equivalent original code set. Four term sets were larger due to deficiencies in the original code sets.

**Discussion:**

Term sets can concisely represent any code set. This may reduce barriers for examining and reusing code sets, which may accelerate research using healthcare databases. We have developed open-source software that supports researchers using term sets.

**Conclusion:**

Term sets are independent of clinical code terminologies and therefore: enable reproducible research; are resistant to terminology changes; and are less error-prone as they are shorter than the equivalent code set.

## Introduction

Clinical code terminologies, such as SNOMED [1] and ICD [2], are dictionaries of terms that allow clinicians to record events in electronic health records (EHRs) using alpha-numeric codes rather than free text. This makes patient records more manageable for clinical care, and allows secondary uses of the data, such as researchers performing retrospective observational studies. Researchers construct clinical codes sets [3–5] to represent the medical concepts they wish to investigate. This is a time-consuming activity, and prone to errors which can lead to biases in subsequent analyses [6]. Storing code sets in a format that facilitates validation, sharing and reuse is important, and called for frequently [7–10].

Code sets, also called code lists and value sets [3,9], range from one code to several thousand. The Value Set Authority Centre (VSAC) [11] provides a repository for code sets allowing their sharing and reuse. Their largest, for “Problem”, contains 117,930 SNOMED codes. This code set is likely not useful, but there are several that are and that contain thousands of codes: Trauma (ICD-10) 18524, Fracture lower body (ICD-10) 5902, Infection (SNOMED) 4066 and Cancer (SNOMED) 3867. Verifying large code sets, by checking that all included codes are correct, and also that no codes are missing, is an enormous task and acts as a barrier to reuse [3]. Updating code sets as terminologies change over time, and sub-setting or extending code sets, are laborious and error-prone activities.

This is important because differences in code sets can cause large variations in findings. Rodriguez et al [12] found rheumatoid arthritis (RA) incidence to be 0.15 per 1000 person-years, while Watson et al [13], in the same database, found it to be 1.03 per 1000 person-years; a sevenfold difference. Another study [14], calculated the weekly incidence of infectious intestinal disease as: 8.3/100,000 if using the World Health Organisation’s ICD-10 code set; 10.24/100,000 if using the Royal College of General Practitioners Research and Surveillance Centre’s ICD-9 code set; and 17.93/100,000 if using the ontological definition on which the paper was based.

The FAIR principles [15] aim to improve the transparency and reusability of scientific data and the algorithms and tools for processing and curating that data. Clinical code sets are a key part of the research process and should abide by FAIR principles; they should be findable, accessible, interoperable and reusable. This is not currently the case. Almost all code sets are unpublished [4] and therefore not accessible. Those that are published, on dedicated repositories such as VSAC or clinicalcodes.org [16], are findable but reuse is a challenge. In theory, reuse is achieved by downloading the relevant code set and applying it to an EHR database. However the task of checking the code set for errors involves reading the definition for each code to confirm that they are correctly in the set, and also speculatively searching the rest of the terminology for codes that may have been omitted. This is arguably as time-consuming as constructing the code set from scratch and is one of the current barriers to reuse. There is also no way currently to determine if a missing code was accidentally or deliberately omitted, therefore impossible to determine if a mistake was made, or if the code set definition contained a subtlety not otherwise described.

## Objective

We propose a new representation of selection criteria for EHR based studies, based on lists of inclusion and exclusion terms. We introduce a methodology for constructing codes sets which takes advantage of this representation, show that our method can represent any possible code set, and in doing so is typically more concise, and therefore practical for other researchers to verify, validate and ultimately reuse with confidence.

## Materials and methods

We introduce ‘term sets’ to define cohort selection criteria for EHR-based studies. A ‘term set’ consists of three parts: inclusion terms describing the feature of interest (e.g. ‘stroke, ‘heart failure’); exclusion terms describing things of no interest (e.g. ‘family history’, ‘screening’); and the target clinical code terminology and version (e.g. terminology = SNOMED-CT, version = uk-edition-v20180401). A code set is created from a term set by searching the terminology for codes that contain inclusion terms but that don’t contain exclusion terms.

### Relationship between code sets and term sets

The traditional representations of cohort selection criteria are clinical code sets which are applied to EHR databases via a query language. Code sets are extensional; they enumerate every code in the set. Term sets by contrast are intensional; they provide necessary and sufficient conditions by which a code is a member of the set. When applied to a particular terminology and version, a term set uniquely defines a code set. For example, consider the phrase “countries of the world” which is intensional, as compared with a complete list of countries of the world which is extensional. The list of countries changes over time, but at any point the intensional set can be derived from the extensional definition. Similarly, the extensional code set can be derived from the intensional term set.

### Procedure for constructing term sets

Our method to construct a term set:

1. Select a clinical code terminology
2. Decide upon one or more inclusion terms, e.g. ‘heart failure’.
3. Perform a search within the terminology for codes with a definition matching the inclusion terms. The search rules are described below.
4. Optionally exclude matching definitions by adding exclusion terms. E.g. for ‘stroke’, it would make sense to exclude the term ‘family history’.
5. For hierarchical code terminologies, return codes that are descendants of matching codes, with definitions that do not contain an inclusion term. Add inclusion or exclusion terms to explicitly include or exclude these descendant codes.
6. Iterate until all inclusion terms have been added, and there are no unmatched descendants.

Deciding upon inclusion and exclusion terms is often a complex task requiring medical expertise. Therefore when implementing this method a clinician would need to be involved, or at the very least an expert in the particular disease domain. However for now we concentrate on the method itself, rather than its implementation. A worked example for the method can be found in S2 Appendix.

### Search rules

#### Case insensitive

The term [fracture] matches “Shoulder fracture” and “Fracture of shoulder”.

#### Words are matched in any order

The term [shoulder fracture] matches “Shoulder fracture” and “Fracture of shoulder”.

#### All words must be present

The term [type 2 diabetes] matches “Diabetes, type 2” and “History of type 2 diabetes”, but not “Type 1 diabetes”.

#### Use quotes to match exactly

The term [“type 2 diabetes”] matches “Type 2 diabetes” and “History of type 2 diabetes” but not “Diabetes, type 2”.

#### Wildcards allow partial word searching

The term [diabet*] matches “Diabetes” and “Diabetic patient”.

#### Exact matches are never excluded

The term [heart failure] always matches “Heart failure” even if [heart] were excluded.

### Proof that any code set can be represented as a term set

This ensures that our method can actually be used in practice for all code sets.

#### Clinical code terminology

A clinical code terminology *T* = (*C*,*D*,*f*) is a set of codes *C*, a set of definitions *D*, and a mapping function *f*:*C*→*D* that links each code *c*∈*C* with a set of one or more definitions *d*∈*D*. Examples for Snomed CT, Read v2 and ICD-10 would be:

$$\begin{array}{l} f_{SnomedCT}(34486009)=\{\left\{\prime Hyperthyroidism\prime ,\prime Hyperthyroidism(disorder)\prime \right\}\}\\ f_{Readv2}(G58..)=\{\prime Heart\;failure\prime ,\prime Cardiac\;failure\prime \}\\ f_{ICD-10}(L71)=\{\prime Rosacea\prime \} \end{array}$$

The mapping function is surjective; each element of *D* is mapped to by at least one element of *C*. The inverse function *f*^−1^:*D*→*C* therefore exists for all definitions in *D* and is defined such that ∀ *d*∈*D*, *f*^−1^(*d*) = *Y* with *c*∈*Y*⇔*d*∈*f*(*c*).

#### Matching definition set

For a set of word sequences *W* = {*w*_1_,…,*w*_*m*_} and a terminology *T* = (*C*,*D*,*f*) we define the matching definition set *MD*(*T*,*W*) as the set of all definitions *d*∈*D* where *w*_*i*_ matches *d*.

$$MD(T,W)={⋃}_{i=1}^{m}MD(T,w_{i})$$ (1)

#### Matching definition set with exclusions

Given two sets of word sequences *W*,*E* and a terminology *T* = (*C*,*D*,*f*) we define the matching definition set with exclusions *MDE*(*T*,*W*,*E*) as the set of all definitions *d*∈*D* where *w*_*i*_ matches *d* and *e*_*j*_ does not match *d*.

$$MDE(T,W,E)=[W\cap D]\cup [MD(T,W)\cap \{MD(T,E){\}}^{C}]$$ (2)

#### Matching concept set

For a terminology *T* = (*C*,*D*,*f*), and two sets of word sequences *W*,*E*, we define the matching concept set *M*(*T*,*W*,*E*) as all codes in the terminology whose definition matches *W*. Alternatively:

$$M(T,W,E)=f^{-1}(MDE(T,W,E))$$ (3)

#### Proposal

Any subset of clinical codes from a terminology can be represented by a set of inclusion terms and a set of exclusion terms. Formally, for terminology *T* = (*C*,*D*,*f*) and any *X* = {*x*_1_,*x*_2_,…,*x*_*n*_}, a subset of *C*, there exists a set of inclusion word sequences *I* = {*i*_1_,*i*_2_,…,*i*_*r*_} and a set of exclusion word sequences *E* = {*e*_1_,*e*_2_,…,*e*_*s*_} such that

M(T,I,E)=X

#### Proof

Let *I* = *f*(*X*) and *E* = *f*(*X*). Then

$$\begin{array}{l} M(T,I,E)=f^{-1}(MDE(T,I,E))\;\mathrm{From}(3)\\ =f^{-1}\left(\left[I\cap D\right]\cup \left[MD\left(T,I\right)\cap {\left\{MD\left(T,E\right)\right\}}^{C}\right]\right)\;\mathrm{From}(2)\\ =f^{-1}\left(\left[f\left(X\right)\cap D\right]\cup \left[MD\left(T,f\left(X\right)\right)\cap {\left\{MD\left(T,f\left(X\right)\right)\right\}}^{C}\right]\right)\;As\;I=f\left(X\right)\;\mathrm{and}\;E=f\left(X\right)\\ =f^{-1}\left(f\left(X\right)\cup \left[MD\left(T,f\left(X\right)\right)\cap {\left\{MD\left(T,f\left(X\right)\right)\right\}}^{C}\right]\right)\;As\;f\left(X\right)\subseteq D\\ =f^{-1}\left(f\left(X\right)\cup \emptyset \right)\;As\;A\cap A^{C}=\emptyset \\ =f^{-1}\left(f\left(X\right)\right)\\ =X\;□ \end{array}$$

For a complete proof and all definitions, see S1 Appendix.

### Term set software

We have developed a web application (https://getset.herokuapp.com) that implements the above methods and allows users to create and verify term sets. The tool is currently implemented for Read v2 codes [17] which are used in UK general practice, however it is straightforward to extend to other hierarchical terminologies like ICD or SNOMED. Once created, term sets can be automatically verified and then shared via GitHub (https://github.com/). Users are encouraged to add their name, a short title and description, so that researchers reusing their set can easily determine their intent.

### Empirical study

The proof above demonstrates “completeness”; any code set can be represented as a term set. We also wished to demonstrate “efficiency”: a term set is shorter than the equivalent code set and is therefore easier and quicker to check. We therefore conducted an empirical study which found published clinical code sets, created their equivalent term set representations, and reported on their relative sizes.

GetSet is currently configured with Read v2, therefore we searched PubMed for papers using the Clinical Practice Research Datalink (CPRD) [18]; a large primary care database containing Read v2 codes with 100s of publications annually. We used the search term ("CPRD"[all fields] or "Clinical Practice Research Datalink"[all fields]) and sorted the results by date descending. Reviewing recent papers ensured we can demonstrate that our method is valid for the current state of the art in clinical code set engineering.

We reviewed each paper in turn and included those that required the construction of code sets to define a cohort of patients. Cohort definition is the focal point of each paper and therefore the code set(s) that are most likely to appear. Also, by focussing on cohort definition, we avoided over-representation from papers with numerous code sets.

For each paper reviewed we extracted any code sets that described a patient cohort for a condition/diagnosis that had not been previously included. Certain conditions will likely be studied more frequently than others; restricting ourselves to one code set per condition ensured we had a sufficient variety of diseases.

We continued to review papers until code sets were discovered from 20 distinct papers. This ensured we would find 20 code sets for a variety of diagnoses and from a variety of authors.

We then created term set representations for each code set, using the above method, with the following caveats:

- Any ‘medcodes’ (CPRD’s code dictionary) were first converted to Read v2 codes.
- We removed all codes except Read v2 (e.g. CPRD also contains Oxmis codes, which were in use pre-2000, and CTV3 codes).
- Where multiple codes have identical definitions, and the code set has included some but not all, we extended the code set to include them all.

For each code set we reported on the code set size and compared this with the number of inclusion and exclusion terms in our equivalent representation.

## Results

The PubMed search was executed on 17^th^ January 2018 by the lead author and returned 809 papers. The target of code sets from 20 distinct papers was reached after reviewing 45 papers; no further papers were reviewed. The 20 papers consisted of: 18 which included their code set in the paper, as a supplement, or in an online repository; 1 with code sets available on request so they were requested and received; and 1 that referenced code sets from another paper so this was retrieved to obtain the code sets. A total of 31 code sets for cohort definitions were found in the 20 papers. For further detail see: https://doi.org/10.5281/zenodo.1316984.

The median number of codes in each code set was 48 (IQR [18,120]). The smallest code set was for Stevens-Johnson syndrome and contained 1 code, while the largest code set, for infections that could lead to a potential hospitalization, contained 3,219 codes.

Each code set was successfully converted into a term set using our previously described procedure. The term sets are available at https://doi.org/10.5281/zenodo.1316984. The full list of code set definitions, their sizes, and the equivalent term set sizes are in Table 1. Nine code sets omitted codes with definitions identical to an included code and so these codes were added prior to the conversion process. As an example, the code set for rheumatoid arthritis included the code “N040R00: Rheumatoid nodule”, but did not include the code “N042200: Rheumatoid nodule”, therefore N042200 was added prior to the conversion to a term set. The full list of extra codes for these nine code sets is available in S1 Table.

**Table 1:** Codes set descriptions and sizes, the size of the related inclusion/exclusion term sets, and the inclusion/exclusion term sizes as proportions of the original code set size. Proportions ≤ 100% are displayed in bold.

Cohort definition code sets	Code set size	Number of inclusion terms	Number of exclusion terms	Number of inclusion and exclusion terms as % of code set size
Type 2 diabetes mellitus [19]	116	5	9	**12.1%**
Cancer except non-melanoma skin cancer [20]	1395	67	144	**15.1%**
Total knee replacement [21]	40	2	8	**25%**
Polymyalgia rheumatic [22]	3	1	0	**33.3%**
Asthma specific [23]	120	4	51	**45.8%**
Hidradenitis suppurativa (HS) [24]	2	1	0	**50%**
Shortness of breath excluded [5]	29	11	4	**51.7%**
Shortness of breath [5]	48	11	14	**52.1%**
Dementia [25]	74	8	31	**52.7%**
Non acute heart failure [26]	40	22	0	**55%**
Ethnicity [27]	183	46	63	**59.6%**
Potential hospitalized infections [28]	3219	1383	537	**59.6%**
Tuberculosis [29]	151	4	95	**65.6%**
Shoulder dislocation [30]	18	2	10	**66.7%**
Country of birth [27]	467	241	88	**70.4%**
Giant cell arteritis [22]	7	4	1	**71.4%**
Type 1 diabetes mellitus [31]	35	4	25	**82.9%**
Psoriatic arthritis [32]	7	5	1	**85.7%**
Possible undiagnosed HS [24]	47	40	2	**89.4**%
Religion [27]	112	72	29	**90.2%**
Fragility fracture [33]	18	3	14	**94.4%**
Rheumatoid arthritis [34]	57	13	42	**96.5%**
Living alone [27]	65	39	25	**98.5%**
Colorectal cancer [35]	23	9	14	**100%**
Stevens-Johnson syndrome [36]	1	1	0	**100%**
Toxic epidermal necrolysis [36]	5	4	1	**100%**
Myotonic dystrophy type 1 [37]	2	2	0	**100%**
Marital status [27]	148	34	131	111.5%
Cohabitation [27]	85	22	79	118.8%
Residence [27]	168	92	111	120.1%
Heart failure [26]	55	20	48	123.6%

The total size of the term sets was on average 74% of the size of the code sets. In four code sets the total number of inclusion and exclusion terms exceeded the size of the code set: marital status, cohabitation, residence and heart failure. The code sets for marital status and cohabitation both use the code “1331.00: Single”. The inclusion term “single” matches many unrelated codes therefore many exclusion terms are needed. The code sets for residence and heart failure were perhaps poorly defined by the original authors. The residence code set aims to include codes that describe a person’s residential status and includes such wonderful terms as “Fall from cliff, occurrence in residential institution” and “Bitten by crocodile, occurrence in residential institution”, but then doesn’t include the terms “Prolonged stay in weightless environment, occurrence in residential institution” or “Victim of avalanche, occurrence in residential institution”. In order to represent this precisely with a term set we needed to include a large number of unnecessary exclusion terms. Finally the heart failure code set includes some, but not all, cardiomyopathy codes. There is no clinical reason for this and the number of inclusion terms would reduce if “cardiomyopathy” could be included, as opposed to the current situation where the exact definition of 15 cardiomyopathy codes must be included.

## Discussion

We have developed a method for creating clinical code sets that incorporates metadata on how the code set was created. We have demonstrated with a formal proof that our method works for any code set, and have shown empirically that the lists of inclusion and exclusion terms are on average shorter than the list of codes themselves.

A recent HL7 initiative provides a method for defining intensional value sets (code sets) [38]. Using this method a researcher can define a set of rules which when applied to a terminology generate a code set. However this does not give the creator of the code set any support, methodology or tools for how to create the rules for the intensional definition. In a similar way, Reference Sets [39] within SNOMED can be used to specify a subset of concepts for use in a particular application, but without creation support. Reference sets are also specific to SNOMED. Our approach provides a generalizable methodology and software tool which are used to build term sets and their associated code sets. Integration of the approaches could be achieved if term sets created with our software were exportable to the HL7 definition of an intensional value set. This would then provide a robust and transparent code set creation process, along with a precise, formal definition.

There are at least four existing tools and associated methodologies for constructing clinical code sets. Davé and Petersen [40] created code sets by searching for synonymous terms and browsing the hierarchy. The final Stata script can be shared so that the process can be scrutinized. Others have developed R/Stata scripts: pcdsearch [41] and CALIBERcodelists [42,43]. These scripts reuse the ideas of Davé and Petersen, while allowing more complex queries using Boolean operators and regular expressions. Recently Watson et al. [5] presented a three-stage process: defining the clinical concept a priori with clinician assistance; searching a clinical terminology using R or Stata to create an initial code set; and producing a final code set via a Delphi exercise with at least two GPs (the main difference to previous approaches).

Our approach builds on the strengths of these methods while addressing certain limitations. Each method above has a way of excluding codes; typically by specifying the codes themselves. By using exclusion terms, we produce metadata that is uncoupled from particular terminologies and is more readable to reviewers of the code set. The output of the above methods is always a script (Stata or R). By not tying our method to a particular scripting language, and using a simple web application, we reduce the barriers to the methodical creation, inspection and reuse of code sets. Allowing regular expressions may help the code set creator, however it will likely act as a further barrier to reuse if the expressions get overcomplicated or if the next researcher is unfamiliar with regular expressions. We have kept our search strategy as simple as possible to mitigate this problem.

Although some of the reviewed code sets may have used one of the above methods, none made available the scripts used to create them. It is probably a safe assumption that this is true for the majority of code sets. The problem, for researchers reusing the code set, is that it is unknown which codes are missing and whether they were omitted deliberately or accidentally. Using our methodology these decisions become explicit. A future researcher may disagree with a decision, but at least it is available for scrutiny, and they can reuse the generated code set by tweaking the definition rather than starting from scratch.

Clinician involvement in code set development is critical, but precisely how research groups incorporate our methodology into their working practices is an open question. One option would be to use the three-stage process from Watson et al. with steps one and two (synonym definition and code set creation) facilitated with our tool.

We found examples where definitions only make sense when considered in the context of the hierarchy. E.g. the term “single” could be a numerical descriptor or a marital status. Our search strategy could be extended to examine the definitions of each codes’ ancestors. A search for “marital status single” would then return the code with the definition “single” only if it had ancestors that contained the words “marital” and “status”. This would alleviate the problem where inclusion terms with low specificity (“single” as a marital status, “white” as an ethnicity) lead to large numbers of exclusion terms.

The Read dictionary has a prefix-based hierarchy (G30’s parent is G3, G3’s parent is G). Two of the code sets we analysed (Dementia and potential hospitalized infections) used wildcards to represent multiple codes, e.g. “A*” to represent “A….” and all of its descendants. This leads to shorter code sets, which are easier to interpret, however it is problematic for two reasons. Firstly, when a code is included in a set it is not necessary that all descendants should also be included, and simply using a wildcard gives no guarantees that the researcher has inspected and accepted each code. Secondly, as the actual codes used in the analysis are not explicitly provided, it is impossible to determine which codes were actually used because code dictionaries change over time, with codes added and removed. Our methodology, which encourages users to specify inclusion (or exclusion) terms to match all descendants of included codes leads to more complete synonym lists and gives extra confidence to researchers reusing the code set.

Various problems were identified in the code sets (examples in Table 2). They fall into three categories: codes are included which do not correspond to the code set description; codes are omitted when they are obviously part of the code set; and some included and omitted codes are contradictory and should either all be included or all omitted. As we aimed to reproduce the code sets exactly, we have invariably created code sets with more inclusion and exclusion terms than are strictly necessary. By correcting the four code sets which had larger associated term sets we saw the average term set to code set proportion fall from 118.5% to 77.3%; all four term sets are now smaller than the code sets. For code sets constructed from scratch using our tool we would expect the number of inclusion and exclusion terms to be further reduced.

**Table 2:** Examples of problems encountered with code sets.

Code set	Example potential problems			Reason for problem
Fragility fracture	Included:	S22..00	Fracture of humerus	The included and omitted codes are contradictory. This leads to additional, unnecessary, inclusion and exclusion terms.
		S222000	Closed fracture of humerus NOS	
	Omitted:	S22z.00	Fracture of humerus NOS	
Potential hospitalized infections	Included:	A53..00	Herpes zoster	The included and omitted codes are contradictory. This leads to additional, unnecessary, inclusion and exclusion terms.
		F501611	Herpes zoster—otitis externa	
		A35..00	Erysipelas	
	Omitted:	F501411	Erysipelas—otitis externa	
	Included:	AB…00	Mycoses (and all descendant codes)	
	Omitted:	FyuN500	Otitis externa in mycoses	
		Hyu0E00	Pneumonia in mycoses classified elsewhere	
		N016.00	Arthropathy associated with mycoses	
Type II diabetes mellitus	Included:	C105100	Diabetes mellitus, adult onset, + ophthalmic manifestation	There is no clinical reason for type II diabetes why you would include the first two codes and exclude the second two. They should all be included.
		C10z100	Diabetes mellitus, adult onset, + unspecified complication	
	Omitted:	C100100	Diabetes mellitus, adult onset, no mention of complication	
		C101100	Diabetes mellitus, adult onset, with ketoacidosis	
Type I diabetes mellitus	Included:	C10EE00	Type 1 diabetes mellitus with hypoglycaemic coma	There is no clinical reason for type I diabetes why you would include the first two codes and exclude the second two. They should all be included.
		C10EN00	Type 1 diabetes mellitus with ketoacidotic coma	
	Omitted:	C10E300	Type 1 diabetes mellitus with multiple complications	
		C10EA00	Type 1 diabetes mellitus without complication	
Rheumatoid arthritis	Included:	N065.00	Unspecified polyarthropathy or polyarthritis	The included and omitted codes are contradictory. This leads to additional, unnecessary, inclusion and exclusion terms.
	Omitted:	N065.11	Polyarthropathy not elsewhere classified	
Marital status	Included:	13IL300	Wife alive	The included and omitted codes are contradictory. This leads to additional, unnecessary, inclusion and exclusion terms.
	Omitted:	13IL700	Husband alive	
	Included:	13IL.00	Health of spouse	
	Omitted:	13Fe.00	Lives with spouse	
	Included:	13ID.00	Partner unemployed	
	Omitted:	13IZ400	Partner alive	
		13IZ500	Partner unwell	
		13IZ600	Partner well	
Cohabitation	Included:	13IL300	Wife alive	The included and omitted codes are contradictory. This leads to additional, unnecessary, inclusion and exclusion terms.
	Omitted:	13IL700	Husband alive	
	Included:	13IL.00	Health of spouse	
	Omitted:	13Fe.00	Lives with spouse	
		13HG.11	Spouse left home	
Living alone	Included:	13FH.00	Lives with relatives	These codes are examples of living with someone and are therefore not examples of living alone.
		13Is.00	Lives with grandfather	
		13It.00	Lives with grandmother	
Residence	Included:	U10F100	Fall from cliff, occurrence in residential institution	The included codes indicate residence in a residential institution. The omitted codes are equivalent to this and should be included.
		U128100	Bitten by crocodile or alligator, occurrence in residential institution	
	Omitted:	U1B2100	Prolonged stay in weightless environment, occurrence in residential inst…	
		U196100	Victim of avalanche, occurrence in residential institution	
Religion	Omitted:	13yL.00	Tibetan Buddhist	These codes are examples of religions and should be included.
	Omitted:	13yu.00	Coptic orthodox	
	Omitted:	13zS.00	Weslyan Methodist	
Country of birth	Omitted:	13dt.00	Born in Isle of Man	These codes are indicative of country of birth and so should be included.
	Omitted:	13du.00	Born in Faroe Islands	
	Omitted:	13dv.00	Born in Greenland	
Ethnicity	Omitted:	9TC..00	Roma ethnic group	These codes are descriptive of ethnicity and so should be included.
	Omitted:	9TC0.00	Bulgarian Roma	
	Omitted:	9TC1.00	Czech Roma	
	Included:	9S6..00	Indian	The included and omitted codes are contradictory. This leads to additional, unnecessary, inclusion and exclusion terms.
	Omitted:	1347.00	Indian origin	
Heart failure	Included:	G55..00	Cardiomyopathy	There is no clinical reason for heart failure why you would include one code for cardiomyopathy but then exclude others. They should all be included.
	Omitted:	G558200	Dystrophic cardiomyopathy	
	Omitted:	G558400	Amyloid cardiomyopathy	
	Omitted:	G558.00	Cardiomyopathy in disease EC	
Shortness of breath	Omitted:	173g.00	Breathlessness causing difficulty eating	This code is a synonym for shortness of breath and so should be included.
Tuberculosis	Included:	Ayu1900	Miliary tuberculosis, unspecified	The included and omitted codes are contradictory. This leads to additional, unnecessary, inclusion and exclusion terms.
	Omitted:	Ayu1800	Other miliary tuberculosis	
	Included:	Ayu1300	Respiratory TB unspecified, no mention of bacteriological confirmation	
	Omitted:	Ayu1100	Respiratory TB unspecified, confirmed bacteriologically and histologically	
Cancer not non-melanoma skin cancer	Omitted:	B305B00	Malignant neoplasm of fourth metacarpal bone	These are types of cancer and should be included.
	Omitted:	ByuB.00	Malignant neoplasm of thyroid and other endocrine glands	
	Omitted:	B640000	B-cell acute lymphoblastic leukaemia	
	Omitted:	B624.12	Hairy cell leukaemia	
	Omitted:	B509.00	Malignant melanoma of eye	
Asthma	Included:	679J000	Health education—asthma self management	The included and omitted codes are contradictory. This leads to additional, unnecessary, inclusion and exclusion terms.
	Omitted:	679J.00	Health education–asthma	

There are reasons why published code sets have omissions that aren’t necessarily errors. A researcher might justifiably decide that it is more important to capture a short list of codes which occur most frequently in their dataset than to focus on codes that occur infrequently or not at all. This may be true for their own research, but for other researchers wanting to reuse their code sets on different data sources it is not good enough. The burden of large code sets might have encouraged researchers to keep their code sets short, but with our methodology this is no longer a restriction, as validation can be performed on the shorter term sets rather than the code sets.

Another valid reason for omissions is that code dictionaries change over time so it is possible that codes recently added to a terminology do not appear in a code set. This becomes a question of how to best keep code sets updated over time, and our approach provides a simple way to do this. Previously when updating a code set a researcher, who hadn’t kept records of their search strategy from several years before, may end up recreating the code set. Now with the inclusion and exclusion terms captured and stored alongside the code set, one simply executes the term set definition against the updated code dictionary to see what additional codes may or may not need to be included.

We have demonstrated our method using Read codes, however the only precondition is that a terminology maps codes to definitions in a hierarchy, so our method would easily transfer to other terminologies such as SNOMED and ICD. One interesting avenue for further investigation is whether code sets can be translated into different terminologies. Once a researcher has defined a code set for one terminology, they could use the web tool to switch to a second terminology and automatically apply the same inclusion and exclusion terms to define a code set for that terminology. This would be useful for researchers using UK primary care data which is migrating from Read to SNOMED.

### Strengths

We have shown that our method works formally via the proof and empirically via the code set mapping exercise. Using recent code sets from a variety of authors and for a variety of conditions demonstrates the generalisability of our technique. We have built upon the ideas from existing tools and methodologies as well as the recommendations from our earlier review [3].

### Limitations

The search for papers was performed by a single author, however given the transparency of the search strategy the biggest risk is that a paper containing a code set has been incorrectly rejected. This would presumably be a random bias and not affect the results. The list of papers reviewed is also available for inspection at https://doi.org/10.5281/zenodo.1316984.

The decision to select code sets for the cohort definition, rather than for the outcomes or the confounders, could have affected the results. However we found code sets for a wide variety of conditions and had few problems converting them into our format, so consider it likely that this would extend to other conditions.

Code sets can be represented in multiple ways, some of which will be easier to understand than others. Some researchers may therefore be able to produce ‘better’ term sets. This can also be seen as a strength, as researchers are more likely to use term sets that are more clearly defined, so these term sets will prevail at the expense of those that are harder to understand.

There may be occasions where it is unclear if a code should be included or not, for example if clinicians use the code in different ways. At present one solution is to create two or more term sets that either include or exclude the uncertain codes. These term sets would have slightly different inclusion and exclusion lists, and their associated description would highlight how sensitive or specific the term set was.

Finally, although largely terminology agnostic, on occasion the particular inclusion and exclusion terms are loosely tied to the terminology used. One extreme example in Read v2 is for the term “G21z00: …without congestive cardic failure” which misspells the word “cardiac”. When selecting this code you would need an inclusion term of “cardic failure” which could be confusing and is unlikely to work in other terminologies. This is, however, an infrequent occurrence.

## Conclusion

We have developed a new representation of cohort selection criteria for EHR based studies, a term set, which consists of: inclusion and exclusion terms; and a clinical code terminology and version. We have described a method to create term sets and developed an open source web application that implements this procedure. We have shown that our representation is as expressive as clinical code sets, but more efficient. Finally, term sets are easier to share, inspect, and reuse, because they are independent of specific (versions of) clinical terminologies. We expect that this will benefit transparent and reproducible research with EHR data.

## Supporting information

S1 Appendix Full proof and definitions. Full formal proof and all definitions for the claim that a term set can represent any code set.
(DOCX)

S1 Table Inconsistent duplicated codes. Codes added to code sets where a code with an identical definition had been excluded.
(DOCX)

S2 Appendix Worked example. Step by step construction of a term set for Type 2 Diabetes.
(DOCX)

## References

[1] SNOMED International. Systematized Nomenclature of Medicine Clinical Terms (SNOMED CT) [Internet].

[2] World Health Organisation. The ICD-10 classification of mental and behavioural disorders: clinical descriptions and diagnostic guidelines Geneva: World Health Organisation; 1992.

[3] Williams R, Kontopantelis E, Buchan I, Peek N. Clinical code set engineering for reusing EHR data for research: A review. J Biomed Inform. 2017;70: 1–13. 10.1016/j.jbi.2017.04.010 28442434

[4] Springate DA, Kontopantelis E, Ashcroft DM, Olier I, Parisi R, Chamapiwa E, et al ClinicalCodes: An online clinical codes repository to improve the validity and reproducibility of research using electronic medical records. PLoS One. 2014;9: e99825 10.1371/journal.pone.0099825 24941260 PMC4062485

[5] Watson J, Nicholson BD, Hamilton W, Price S. Identifying clinical features in primary care electronic health record studies: methods for codelist development. BMJ Open. British Medical Journal Publishing Group; 2017;7: e019637 10.1136/bmjopen-2017-019637 29170293 PMC5719324

[6] Gulliford MC, Charlton J, Ashworth M, Rudd AG, Toschke AM, Delaney B, et al Selection of medical diagnostic codes for analysis of electronic patient records. Application to stroke in a primary care database. PLoS One. 2009;4 10.1371/journal.pone.0007168 19777060 PMC2744876

[7] Muller S, Hider SL, Raza K, Stack RJ, Hayward RA, Mallen CD. An algorithm to identify rheumatoid arthritis in primary care: a Clinical Practice Research Datalink study. BMJ Open. 2015;5: e009309 10.1136/bmjopen-2015-009309 26700281 PMC4691776

[8] Rañopa M, Douglas I, van Staa T, Smeeth L, Klungel O, Reynolds R, et al The identification of incident cancers in UK primary care databases: a systematic review. Pharmacoepidemiol Drug Saf. 2015;24: 11–18. 10.1002/pds.3729 25421570

[9] Mo H, Thompson WK, Rasmussen L V, Pacheco JA, Jiang G, Kiefer R, et al Desiderata for computable representations of electronic health records-driven phenotype algorithms. J Am Med Inform Assoc. 2015;22: 1220–30. 10.1093/jamia/ocv112 26342218 PMC4639716

[10] Winnenburg R, Bodenreider O. Metrics for assessing the quality of value sets in clinical quality measures. AMIA Annu Symp Proc. 2013;2013: 1497–1505. Available: http://www.scopus.com/inward/record.url?eid=2-s2.0-84901264877&partnerID=40&md5=b556126c6cc1285aab43d87b63ec7ae9 24551422 PMC3900160

[11] Bodenreider O, Nguyen D, Chiang P, Chuang P, Madden M, Winnenburg R, et al The NLM value set authority center. Stud Health Technol Inform. 2013;192: 1224 10.3233/978-1-61499-289-9-1224 23920998 PMC4300102

[12] Rodríguez LAG, Tolosa LB, Ruigómez A, Johansson S, Wallander M-A. Rheumatoid arthritis in UK primary care: incidence and prior morbidity. Scand J Rheumatol. 38: 173–7. 10.1080/03009740802448825 19117247

[13] Watson DJ, Rhodes T, Guess HA. All-cause mortality and vascular events among patients with rheumatoid arthritis, osteoarthritis, or no arthritis in the UK General Practice Research Database. J Rheumatol. 2003;30: 1196–202. Available: http://www.ncbi.nlm.nih.gov/pubmed/12784389 12784389

[14] de Lusignan S, Shinneman S, Yonova I, van Vlymen J, Elliot AJ, Bolton F, et al An Ontology to Improve Transparency in Case Definition and Increase Case Finding of Infectious Intestinal Disease: Database Study in English General Practice. JMIR Med Informatics. JMIR Medical Informatics; 2017;5: e34 10.2196/medinform.7641 28958989 PMC5639210

[15] Wilkinson MD, Dumontier M, Aalbersberg IjJ, Appleton G, Axton M, Baak A, et al The FAIR Guiding Principles for scientific data management and stewardship. Sci Data. 2016;3: 160018 10.1038/sdata.2016.18 26978244 PMC4792175

[16] Springate D, Kontopantelis E, Ashcroft D, Olier I, Parisi R, Chamapiwa E, et al. ClinicalCodes.org [Internet]. [cited 1 Mar 2016]. Available: https://clinicalcodes.rss.mhs.man.ac.uk/

[17] Chisholm J. The Read Clinical Classification. Health Bull (Raleigh). 1990;50: 422–427. 10.1136/bmj.300.6732.1092 1483867

[18] Herrett E, Gallagher AM, Bhaskaran K, Forbes H, Mathur R, Staa T van, et al Data Resource Profile: Clinical Practice Research Datalink (CPRD). Int J Epidemiol. 2015;44: 827–836. 10.1093/ije/dyv098 26050254 PMC4521131

[19] Spanopoulos D, Barrett B, Busse M, Roman T, Poole C. Prescription of DPP-4 Inhibitors to Type 2 Diabetes Mellitus Patients With Renal Impairment: A UK Primary Care Experience. Clin Ther. Elsevier; 2018;40: 152–154. 10.1016/j.clinthera.2017.11.009 29246708

[20] Busby J, McMenamin Ú, Spence A, Johnston BT, Hughes C, Cardwell CR, et al Angiotensin receptor blocker use and gastro-oesophageal cancer survival: a population-based cohort study. Aliment Pharmacol Ther. 2018;47: 279–288. 10.1111/apt.14388 29105106

[21] Khan T, Alvand A, Prieto-Alhambra D, Culliford DJ, Judge A, Jackson WF, et al ACL and meniscal injuries increase the risk of primary total knee replacement for osteoarthritis: a matched case–control study using the Clinical Practice Research Datalink (CPRD). Br J Sports Med. 2018; bjsports-2017-097762. 10.1136/bjsports-2017-097762 29331994

[22] Paskins Z, Whittle R, Sultan AA, Muller S, Blagojevic-Bucknall M, Helliwell T, et al Risk of fracture among patients with polymyalgia rheumatica and giant cell arteritis: A population-based study. BMC Med. 2018;16: 4 10.1186/s12916-017-0987-1 29316928 PMC5761155

[23] Young GJ, Harrison S, Turner EL, Walsh EI, Oliver SE, Ben-Shlomo Y, et al Prostate-specific antigen (PSA) testing of men in UK general practice: A 10-year longitudinal cohort study. BMJ Open. British Medical Journal Publishing Group; 2017;7: e017729 10.1136/bmjopen-2017-017729 29084797 PMC5665300

[24] Ingram JRR, Jenkins-Jones S, Knipe DWW, Morgan CLILI, Cannings-John R, Piguet V. Population-based Clinical Practice Research Datalink study using algorithm modelling to identify the true burden of hidradenitis suppurativa. Br J Dermatol. 2018;178: 917–924. 10.1111/bjd.16101 29094346

[25] Donegan K, Fox N, Black N, Livingston G, Banerjee S, Burns A. Trends in diagnosis and treatment for people with dementia in the UK from 2005 to 2015: a longitudinal retrospective cohort study. Lancet Public Heal. Elsevier; 2017;2: e149–e156. 10.1016/S2468-2667(17)30031-2 29253388

[26] Conrad N, Judge A, Tran J, Mohseni H, Hedgecott D, Crespillo AP, et al Temporal trends and patterns in heart failure incidence: A population-based study of 4 million individuals. The Lancet. Elsevier; 10 2 2017: 572–580. 10.1016/S1474-4422(17)30162-X 29174292 PMC5814791

[27] Jain A, van Hoek AJ, Walker JL, Mathur R, Smeeth L, Thomas SL. Identifying social factors amongst older individuals in linked electronic health records: An assessment in a population based study. Forloni G, editor. PLoS One. Public Library of Science; 2017;12: e0189038 10.1371/journal.pone.0189038 29190680 PMC5708811

[28] Saine ME, Gizaw M, Carbonari DM, Newcomb CW, Roy JA, Cardillo S, et al Validity of diagnostic codes to identify hospitalizations for infections among patients treated with oral anti-diabetic drugs. Pharmacoepidemiology and Drug Safety. 18 12 2017 10.1002/pds.4368 29250905

[29] Yates TA, Tomlinson LA, Bhaskaran K, Langan S, Thomas S, Smeeth L, et al Lansoprazole use and tuberculosis incidence in the United Kingdom Clinical Practice Research Datalink: A population based cohort. Metcalfe JZ, editor. PLoS Med. Public Library of Science; 2017;14: e1002457 10.1371/journal.pmed.1002457 29161254 PMC5697821

[30] Shah A, Judge A, Delmestri A, Edwards K, Arden NK, Prieto-Alhambra D, et al Incidence of shoulder dislocations in the UK, 1995–2015: a population-based cohort study. BMJ Open. British Medical Journal Publishing Group; 2017;7: e016112 10.1136/bmjopen-2017-016112 29138197 PMC5695490

[31] Ferreira GLC, Marano C, De Moerlooze L, Guignard A, Feng Y, El Hahi Y, et al Incidence and prevalence of hepatitis B in patients with diabetes mellitus in the UK: A population-based cohort study using the UK Clinical Practice Research Datalink. J Viral Hepat. 2018;25: 571–580. 10.1111/jvh.12841 29220868

[32] Charlton R, Green A, Shaddick G, Snowball J, Nightingale A, Tillett W, et al Risk of uveitis and inflammatory bowel disease in people with psoriatic arthritis: A population-based cohort study. Ann Rheum Dis. BMJ Publishing Group Ltd; 2018;77: 277–280. 10.1136/annrheumdis-2017-212328 29092855

[33] Gamble J-MM, Donnan JR, Chibrikov E, Twells LK, Midodzi WK, Majumdar SR. The risk of fragility fractures in new users of dipeptidyl peptidase-4 inhibitors compared to sulfonylureas and other anti-diabetic drugs: A cohort study. Diabetes Res Clin Pract. Elsevier; 2018;136: 159–167. 10.1016/j.diabres.2017.12.008 29258886

[34] Judge A, Garriga C, Arden NK, Lovestone S, Prieto-Alhambra D, Cooper C, et al Protective effect of antirheumatic drugs on dementia in rheumatoid arthritis patients. Alzheimer’s Dement Transl Res Clin Interv. Elsevier; 2017;3: 612–621. 10.1016/j.trci.2017.10.002 29201995 PMC5700830

[35] Khosrow-Khavar F, Yin H, Barkun A, Bouganim N, Azoulay L. Aromatase inhibitors and the risk of colorectal cancer in postmenopausal women with breast cancer. Ann Oncol. 2018;29: 744–748. 10.1093/annonc/mdx822 29293897 PMC5889043

[36] Frey N, Bircher A, Bodmer M, Jick SS, Meier CR, Spoendlin J. Antibiotic Drug Use and the Risk of Stevens-Johnson Syndrome and Toxic Epidermal Necrolysis: A Population-Based Case-Control Study. J Invest Dermatol. 2018;138: 1207–1209. 10.1016/j.jid.2017.12.015 29273314

[37] Wang Y, Pfeiffer RM, Alsaggaf R, Meeraus W, Gage JC, Anderson LA, et al Risk of skin cancer among patients with myotonic dystrophy type 1 based on primary care physician data from the U.K. Clinical Practice Research Datalink. Int J Cancer. 2018;142: 1174–1181. 10.1002/ijc.31143 29114849 PMC5773358

[38] HL7.org. FHIR v3.0.1—ValueSet [Internet]. [cited 23 Oct 2018]. Available: https://www.hl7.org/fhir/valueset.html

[39] SNOMED International. Reference Sets [Internet]. [cited 23 Oct 2018]. Available: https://confluence.ihtsdotools.org/display/DOCTIG/3.2.1.+Reference+Sets

[40] Dave S, Petersen I. Creating medical and drug code lists to identify cases in primary care databases. Pharmacoepidemiol Saf. 2009;18: 704–707. 10.1002/pds.1770 19455565

[41] Olier I, Springate DA, Ashcroft DM, Doran T, Reeves D, Planner C, et al Modelling conditions and health care processes in Electronic Health Records: an application to Severe Mental Illness with the Clinical Practice Research Datalink. PLoS One. 2015;11: e0146715 10.1371/journal.pone.0146715 26918439 PMC4769302

[42] Shah A. CALIBERcodelists user guide [Internet]. 2014. Available: https://r-forge.r-project.org/scm/viewvc.php/*checkout*/pkg/CALIBERcodelists/inst/doc/userguide.pdf?root=caliberanalysis

[43] Denaxas SC, George J, Herrett E, Shah AD, Kalra D, Hingorani AD, et al Data resource profile: Cardiovascular disease research using linked bespoke studies and electronic health records (CALIBER). Int J Epidemiol. 2012;41: 1625–1638. 10.1093/ije/dys188 23220717 PMC3535749

