# Supplementary material for: Term sets: A transparent and reproducible representation of clinical code sets
Source: PLoS One. 2019 Feb 14;14(2):e0212291. doi: 10.1371/journal.pone.0212291 (PMC6375602; doi:10.1371/journal.pone.0212291)
Supplement: S1 Appendix — Full formal proof and all definitions for the claim that a term set can represent any code set. (DOCX) [file pone.0212291.s001.docx]

S1 Appendix – Full proof and definitions

Definitions and notation

Note on character sets

For simplicity the below assumes that all definitions in a terminology only contain characters in ASCII[42]. In practice this is likely true for most languages using the Latin alphabet, with the exception that there are no accented characters (e.g. é, ö, ç) in ASCII. The logic and proof below remains valid with any alphabet.

Definition – “clinical code” and “clinical code definition”

Let$A$ be the alphabet of printable ASCII characters i.e. those with a decimal value between 32 and 126 inclusive. A clinical code$c$ is a finite sequence of printable ASCII characters:

|  |  |  |
| --- | --- | --- |
|  | $c=c_{1}c_{2}\ldots c_{m} with m<\infty and c_{i}\in A \forall i\in\left[ 1,m \right]$ | (1) |
|  |  |  |

A clinical code definition$d$ is defined equivalently:

|  |  |  |
| --- | --- | --- |
|  | $d=d_{1}d_{2}\ldots d_{n} with n<\infty and d_{i}\in A \forall i\in\left[ 1,n \right]$ | (2) |
|  |  |  |

In reality clinical codes (hereafter simply called codes) are typically shorter than clinical code definitions (hereafter called definitions), and definitions are readable in a human language, while codes are not. However the following proofs do not require these restrictions.

Definition - “clinical code terminology”

A clinical code terminology $T=\left( C,D,f \right)$ is defined by a set of codes$C$, a set of definitions$D$, and a mapping function $f:C\to D$ that links each code $c\in C$ with a set of one or more definitions$d\in D$. Examples for SNOMED CT, Read v2 and ICD-10 would be:

$$f_{SnomedCT}\left( 34486009 \right)=\left\{ \left\{ 'Hyperthyroidism', 'Hyperthyroidism (disorder\mathbf{)'} \right\} \right\}$$

$f_{Readv2}\left( G65.. \right)={\{}^{'}Transient cerebral ischaemia^{'},^{'}Transient ischaemic attack^{'},^{'}Drop attack^{'}\}$

$$f_{ICD-10}\left( L71 \right)=\left\{ '\mathrm{Rosacea}' \right\}$$

The definition for each code can have multiple synonymous definitions and potentially translations into other languages.

The mapping function is surjective, i.e. each element of$D$ is mapped to by at least one element of$C$. The inverse function$f^{-1}:D\to C$ therefore exists for all definitions in$D$ and is defined such that$\forall d\in D, f^{-1}\left( d \right)=Y$ with$c\in Y\Leftrightarrow d\in f\left( c \right)$.

Notation

Given a terminology$T=\left( C,D,f \right)$, and $X=\left\{ x_{1},x_{2},...,x_{n} \right\}$a subset of$C$, then we define $f\left( X \right)=Y$ where $Y=\bigcup_{i=1}^{n} Y_{i}$is the set of definitions such that $f\left( x_{i} \right)=Y_{i}$for$i\in\left[ 1,n \right]$. That is:

|  |  |  |
| --- | --- | --- |
|  | $f\left( X \right)=f\left( \bigcup_{i=1}^{n} \left\{ x_{i} \right\} \right)=\bigcup_{i=1}^{n} f\left( x_{i} \right)=\bigcup_{i=1}^{n} Y_{i}=Y$ | (3) |
|  |  |  |

For$Y=\left\{ y_{1},y_{2},...,y_{m} \right\}$a subset of$D$ we can also define $f^{-1}\left( Y \right)=Z$ where$Z=\bigcup_{i=1}^{m} Z_{i}$is the set of codes such that $f^{-1}\left( y_{i} \right)=Z_{i}$for$i\in\left[ 1,m \right]$. That is:

|  |  |  |
| --- | --- | --- |
|  | $f^{-1}\left( Y \right)=f^{-1}\left( \bigcup_{i=1}^{m} \left\{ y_{i} \right\} \right)=\bigcup_{i=1}^{m} f^{-1}\left( y_{i} \right)=\bigcup_{i=1}^{m} Z_{i}=Z$ | (4) |
|  |  |  |

Assumption

We assume that the inverse function is a one-to-one mapping. That is $\forall c\in C$ let$Y=f\left( c \right)$ then for$d\in D, f^{-1}\left( d \right)=\left\{ c \right\}\Leftrightarrow d\in Y$.

Lemma

Given a terminology$T=\left( C,D,f \right)$, and a subset of$C$, $A=\left\{ a_{1},a_{2},...,a_{r} \right\}$ we show$f^{-1}\left( f\left( A \right) \right)=A$.

Proof

$$f^{-1}\left( f\left( A \right) \right)=f^{-1}\left( f\left( \bigcup_{i=1}^{r} a_{i} \right) \right)$$

$=f^{-1}\left( \bigcup_{i=1}^{r} f\left( a_{i} \right) \right)$ From (3)

$=f^{-1}\left( \bigcup_{i=1}^{r} Y_{i} \right)$ By letting$f\left( a_{i} \right)=Y_{i}$

$=\bigcup_{i=1}^{r} f^{-1}\left( Y_{i} \right)$ From (4)

$=\bigcup_{i=1}^{r} f^{-1}\left( \bigcup_{y\in Y_{i}} y \right)$

$=\bigcup_{i=1}^{r} \bigcup_{y\in Y_{i}} f^{-1}\left( y \right)$ From (4)

$=\bigcup_{i=1}^{r} \bigcup_{y\in Y_{i}} \left\{ a_{i} \right\}$ From assumption 1.1.5

$$=\bigcup_{i=1}^{r} a_{i}$$

$=A$ ⬜

Definition – “word boundary”

Given a definition$d=d_{1}d_{2}\ldots d_{n}$, we define$\alpha$ as the empty character at the start of the definition, and$\omega$ as the empty character at the end of the definition. Our definition is now$d=\alpha d_{1}d_{2}\ldots d_{n}\omega$. If$N$ is the set of non-alphanumeric ASCII characters, then we define a word boundary as any character in the set$N\cup\left\{ \alpha,\omega\right\}$.

Definition – “word” and “word sequence”

Given a definition$d=d_{0}d_{1}d_{2}\ldots d_{n}d_{n+1} \left( d_{0}=\alpha,d_{n+1}=\omega\right)$, we define a word$w=w_{1}w_{2}\ldots w_{m}$ as an alphanumeric contiguous subsequence surrounded by word boundaries. Or formally:$\exists j\in\left[ 0,n-m \right] st \forall i\in\left[ 1,m \right]$ $w_{i}=d_{j+i}$ is not a word boundary and$d_{j}$ and$d_{j+m+1}$ are both word boundaries.

The definition for a word sequence is the same except each$w_{i}$, apart from $w_{0}$ and$w_{m}$, can now be a word boundary. A word is therefore a word sequence that does not contain a word boundary.

Notation

Given a definition$d$, let$d^{*}$ be the set of all word sequences contained within$d$.

Definition - “matching” and “exact matching”

We say a word sequence$w$ matches a definition$d\Leftrightarrow w\in d^{*}$. If$w=d$ we say it is an exact match.

Definition - “matching definition set”

Given a word sequence$w$ and a terminology $T=\left( C,D,f \right)$ we define the matching definition set $MD\left( T,w \right)$ as the set of all definitions $d\in D$ where $w$ matches$d$.

For a set of $m$ word sequences$W=\left\{ w_{1},...,w_{m} \right\}$, we define:

|  |  |  |
| --- | --- | --- |
|  | $MD\left( T,W \right)=\bigcup_{i=1}^{m} MD\left( T,w_{i} \right)$ | (5) |
|  |  |  |

Definition - “matching definition set with exclusions”

Given two word sequences$w,e$ and a terminology $T=\left( C,D,f \right)$ we define the matching definition set with exclusions $MDE\left( T,w,e \right)$ as the set of all definitions $d\in D$ where $w$ matches$d$ and $e$ does not match$d$. If $w$ exactly matches$d$, then$d\in MDE\left( T,w,e \right)$ even if $e$ matches$d$.

|  |  |  |
| --- | --- | --- |
|  | $MDE\left( T,w,e \right)=\left[ w\cap D \right]\cup\left[ MD\left( T,w \right)\cap\left\{ MD\left( T,e \right) \right\}^{C} \right]$ | (6) |
|  |  |  |

For a set of $m$ word sequences$W=\left\{ w_{1},...,w_{m} \right\}$, and a set of $n$ exclusion word sequences$E=\left\{ e_{1},...,e_{n} \right\}$, we define:

|  |  |  |
| --- | --- | --- |
|  | $MDE\left( T,W,E \right)=\left[ W\cap D \right]\cup\left[ MD\left( T,W \right)\cap\left\{ MD\left( T,E \right) \right\}^{C} \right]$ | (7) |
|  |  |  |

Definition - “matching concept set”

For a terminology$T=\left( C,D,f \right)$, and word sequences$w,e$, we define the matching concept set $M\left( T,w,e \right)$ as all codes in the terminology whose definition matches$w$. Alternatively:

|  |  |  |
| --- | --- | --- |
|  | $M\left( T,w,e \right)=f^{-1}\left( MDE\left( T,w,e \right) \right)$ | (8) |
|  |  |  |

For a set of $m$ word sequences$W=\left\{ w_{1},...,w_{m} \right\}$ we define:

|  |  |  |
| --- | --- | --- |
|  | $M\left( T,W,E \right)=f^{-1}\left( MDE\left( T,W,E \right) \right)$ | (9) |
|  |  |  |

Main proposal

Proposal

Any subset of clinical codes taken from a terminology can be represented by a set of inclusion terms and a set of exclusion terms. Formally, given a terminology $T=\left( C,D,f \right)$ and any$X=\left\{ x_{1},x_{2},...,x_{n} \right\}$, a subset of$C$, there exists a set of inclusion word sequences $I=\left\{ i_{1},i_{2},...,i_{r} \right\}$ and a set of exclusion word sequences$E=\left\{ e_{1},e_{2},...,e_{s} \right\}$ such that

|  |  |  |
| --- | --- | --- |
|  | $M\left( T,I,E \right)=X$ | (10) |
|  |  |  |

Proof

Let$I=f\left( X \right)$ and$E=f\left( X \right)$. Then

$M\left( T,I,E \right)=f^{-1}\left( MDE\left( T,I,E \right) \right)$ From (9)

$=f^{-1}\left( \left[ I\cap D \right]\cup\left[ MD\left( T,I \right)\cap\left\{ MD\left( T,E \right) \right\}^{C} \right] \right)$ From (7)

$=f^{-1}\left( \left[ f\left( X \right)\cap D \right]\cup\left[ MD\left( T,f\left( X \right) \right)\cap\left\{ MD\left( T,f\left( X \right) \right) \right\}^{C} \right] \right)$ As$I=f\left( X \right)$ and$E=f\left( X \right)$

$=f^{-1}\left( f\left( X \right)\cup\left[ MD\left( T,f\left( X \right) \right)\cap\left\{ MD\left( T,f\left( X \right) \right) \right\}^{C} \right] \right)$ As$f\left( X \right)\subseteq D$

$=f^{-1}\left( f\left( X \right)\cup\emptyset\right)$ As$A\cap A^{C}=\emptyset$

$=f^{-1}\left( f\left( X \right) \right)$

$=X$ ⬜ From lemma 1.1.6

Assumption revisited

If we drop the assumption that$f^{-1}:D\to C$ is a one-to-one function then we no longer have$f^{-1}\left( f\left( X \right) \right)=X$ from lemma 1.1.6. Instead $f^{-1}\left( f\left( X \right) \right)=X\cup Y$ where$y\in Y$ are codes not in$X$ but with a definition identical to a code in$X$. Therefore our approach does not work$\forall X\subseteq C$ such that$f^{-1}\left( f\left( X \right) \right)=X\cup Y$ where$Y\neq\emptyset$. Or, less formally, if two codes have identical definitions then it is impossible to have a code set including one code, but excluding the other.

We would argue that a terminology should not have multiple codes with the same definition. However it does happen such as in Read v2 where 177..00, SM7y200 and SM7z.11 all have the definition 'Smoke inhalation', and N064K11, N094513, N096512, N220Y00 all have the definition 'Irritable hip'.

Given two distinct codes, x and y, with identical definitions there are two possible scenarios:

1. x and y represent identical concepts and should therefore either both be in a code set, or both be absent from a code set.
2. x and y represent non-identical concepts, in which case the terminology is at fault and the definitions should be changed to ensure that users of the terminology can distinguish between these two concepts

S1 Table - Codes added to code sets due to having identical definitions to already included codes

| Code set | Codes added to initial code set |
| --- | --- |
| Potential hospitalized infections | F0...00 Inflammatory diseases of the central nervous system  F03X.00 Bacterial meningoencephalitis and meningomyelitis, not elsewhere classified  Gy0..00 Cardiovascular syphilis  Hyu0000 [X]Other acute sinusitis  Hyu0900 [X]Pneumonia due to other aerobic gram-negative bacteria  Hyu0A00 [X]Other bacterial pneumonia  Hyu1100 [X]Acute bronchiolitis due to other specified organisms  Hyu2200 [X]Other chronic sinusitis  J154400 Helicobacter gastritis  Kyu5000 [X]Other chronic cystitis  Kyu5100 [X]Other cystitis  M102.11 Pustular eczema  N018.00 Tuberculous arthritis |
| Type II diabetes mellitus | C109311 Type II diabetes mellitus with multiple complications  C109312 Type 2 diabetes mellitus with multiple complications  C109911 Type II diabetes mellitus without complication  C109912 Type 2 diabetes mellitus without complication  C109A12 Type 2 diabetes mellitus with mononeuropathy  C109B12 Type 2 diabetes mellitus with polyneuropathy  C109F00 Non-insulin-dependent diabetes mellitus with peripheral angiopathy  C109H00 Non-insulin dependent diabetes mellitus with neuropathic arthropathy  C10F111 Type II diabetes mellitus with ophthalmic complications  C10F211 Type II diabetes mellitus with neurological complications  C10F511 Type II diabetes mellitus with gangrene  C10FA11 Type II diabetes mellitus with mononeuropathy  C10FC11 Type II diabetes mellitus with nephropathy  C10FD11 Type II diabetes mellitus with hypoglycaemic coma  C10FF11 Type II diabetes mellitus with peripheral angiopathy  C10FG11 Type II diabetes mellitus with arthropathy  C10FH11 Type II diabetes mellitus with neuropathic arthropathy  C10FK11 Hyperosmolar non-ketotic state in type II diabetes mellitus  C10FN11 Type II diabetes mellitus with ketoacidosis  C10FP11 Type II diabetes mellitus with ketoacidotic coma  C10FQ11 Type II diabetes mellitus with exudative maculopathy  C10FR11 Type II diabetes mellitus with gastroparesis |
| Type I diabetes mellitus | C108711 Type I diabetes mellitus with retinopathy  C108712 Type 1 diabetes mellitus with retinopathy  C108911 Type I diabetes mellitus maturity onset  C108912 Type 1 diabetes mellitus maturity onset  C108E11 Type I diabetes mellitus with hypoglycaemic coma  C108E12 Type 1 diabetes mellitus with hypoglycaemic coma  C10E.11 Type I diabetes mellitus  C10E712 Insulin dependent diabetes mellitus with retinopathy  C10E911 Type I diabetes mellitus maturity onset  C10E912 Insulin dependent diabetes maturity onset  C10EE00 Type 1 diabetes mellitus with hypoglycaemic coma  C10EE11 Type I diabetes mellitus with hypoglycaemic coma  C10EE12 Insulin dependent diabetes mellitus with hypoglycaemic coma  C10EK11 Type I diabetes mellitus with persistent proteinuria  C10EL11 Type I diabetes mellitus with persistent microalbuminuria  C10EM11 Type I diabetes mellitus with ketoacidosis  C10EN11 Type I diabetes mellitus with ketoacidotic coma |
| Rheumatoid arthritis | N042200 Rheumatoid nodule |
| Living alone | 13Fc.00 Lives alone  ZV60015 [V]Vagabond |
| Residence | 8Hs..00 Discharge to nursing home  ZV60015 [V]Vagabond |
| Religion | 135a.11 Moravian  13zE.11 United Reformed Church |
| Shoulder dislocation | N083G00 Recurrent dislocation of shoulder - anterior  N083H00 Recurrent subluxation of shoulder - anterior  S410y00 Other closed traumatic dislocation of shoulder  S410z00 Closed traumatic dislocation of shoulder not otherwise specified  S412000 Closed traumatic subluxation shoulder joint  S412z00 Closed traumatic subluxation shoulder NOS |
| Cancer except non-melanoma skin cancer | B592.00 Malignant neoplasms of independent (primary) multiple sites  B592X00 Kaposi's sarcoma of multiple organs  B627B00 Other types of follicular non-Hodgkin's lymphoma  B628.00 Follicular lymphoma  B630011 Extramedullary plasmacytoma  B73..12 Osteoma  B73z.11 Chondroma  B911013 Choriocarcinoma  B936.12 Plasmacytoma NOS  B937.13 Megakaryocytic myelosclerosis  ByuB000 [X]Malignant neoplasm - pluriglandular involvement, unspecified |
