# Supplementary material for: Term sets: A transparent and reproducible representation of clinical code sets
Source: PLoS One. 2019 Feb 14;14(2):e0212291. doi: 10.1371/journal.pone.0212291 (PMC6375602; doi:10.1371/journal.pone.0212291)
Supplement: S1 Table — Codes added to code sets where a code with an identical definition had been excluded. (DOCX) [file pone.0212291.s002.docx]

Appendix

Table S2 - Codes added to code sets due to having identical definitions to already included codes

| Code set | Codes added to initial code set |
| --- | --- |
| Potential hospitalized infections | F0...00 Inflammatory diseases of the central nervous system  F03X.00 Bacterial meningoencephalitis and meningomyelitis, not elsewhere classified  Gy0..00 Cardiovascular syphilis  Hyu0000 [X]Other acute sinusitis  Hyu0900 [X]Pneumonia due to other aerobic gram-negative bacteria  Hyu0A00 [X]Other bacterial pneumonia  Hyu1100 [X]Acute bronchiolitis due to other specified organisms  Hyu2200 [X]Other chronic sinusitis  J154400 Helicobacter gastritis  Kyu5000 [X]Other chronic cystitis  Kyu5100 [X]Other cystitis  M102.11 Pustular eczema  N018.00 Tuberculous arthritis |
| Type II diabetes mellitus | C109311 Type II diabetes mellitus with multiple complications  C109312 Type 2 diabetes mellitus with multiple complications  C109911 Type II diabetes mellitus without complication  C109912 Type 2 diabetes mellitus without complication  C109A12 Type 2 diabetes mellitus with mononeuropathy  C109B12 Type 2 diabetes mellitus with polyneuropathy  C109F00 Non-insulin-dependent diabetes mellitus with peripheral angiopathy  C109H00 Non-insulin dependent diabetes mellitus with neuropathic arthropathy  C10F111 Type II diabetes mellitus with ophthalmic complications  C10F211 Type II diabetes mellitus with neurological complications  C10F511 Type II diabetes mellitus with gangrene  C10FA11 Type II diabetes mellitus with mononeuropathy  C10FC11 Type II diabetes mellitus with nephropathy  C10FD11 Type II diabetes mellitus with hypoglycaemic coma  C10FF11 Type II diabetes mellitus with peripheral angiopathy  C10FG11 Type II diabetes mellitus with arthropathy  C10FH11 Type II diabetes mellitus with neuropathic arthropathy  C10FK11 Hyperosmolar non-ketotic state in type II diabetes mellitus  C10FN11 Type II diabetes mellitus with ketoacidosis  C10FP11 Type II diabetes mellitus with ketoacidotic coma  C10FQ11 Type II diabetes mellitus with exudative maculopathy  C10FR11 Type II diabetes mellitus with gastroparesis |
| Type I diabetes mellitus | C108711 Type I diabetes mellitus with retinopathy  C108712 Type 1 diabetes mellitus with retinopathy  C108911 Type I diabetes mellitus maturity onset  C108912 Type 1 diabetes mellitus maturity onset  C108E11 Type I diabetes mellitus with hypoglycaemic coma  C108E12 Type 1 diabetes mellitus with hypoglycaemic coma  C10E.11 Type I diabetes mellitus  C10E712 Insulin dependent diabetes mellitus with retinopathy  C10E911 Type I diabetes mellitus maturity onset  C10E912 Insulin dependent diabetes maturity onset  C10EE00 Type 1 diabetes mellitus with hypoglycaemic coma  C10EE11 Type I diabetes mellitus with hypoglycaemic coma  C10EE12 Insulin dependent diabetes mellitus with hypoglycaemic coma  C10EK11 Type I diabetes mellitus with persistent proteinuria  C10EL11 Type I diabetes mellitus with persistent microalbuminuria  C10EM11 Type I diabetes mellitus with ketoacidosis  C10EN11 Type I diabetes mellitus with ketoacidotic coma |
| Rheumatoid arthritis | N042200 Rheumatoid nodule |
| Living alone | 13Fc.00 Lives alone  ZV60015 [V]Vagabond |
| Residence | 8Hs..00 Discharge to nursing home  ZV60015 [V]Vagabond |
| Religion | 135a.11 Moravian  13zE.11 United Reformed Church |
| Shoulder dislocation | N083G00 Recurrent dislocation of shoulder - anterior  N083H00 Recurrent subluxation of shoulder - anterior  S410y00 Other closed traumatic dislocation of shoulder  S410z00 Closed traumatic dislocation of shoulder not otherwise specified  S412000 Closed traumatic subluxation shoulder joint  S412z00 Closed traumatic subluxation shoulder NOS |
| Cancer except non-melanoma skin cancer | B592.00 Malignant neoplasms of independent (primary) multiple sites  B592X00 Kaposi's sarcoma of multiple organs  B627B00 Other types of follicular non-Hodgkin's lymphoma  B628.00 Follicular lymphoma  B630011 Extramedullary plasmacytoma  B73..12 Osteoma  B73z.11 Chondroma  B911013 Choriocarcinoma  B936.12 Plasmacytoma NOS  B937.13 Megakaryocytic myelosclerosis  ByuB000 [X]Malignant neoplasm - pluriglandular involvement, unspecified |
