# Supplementary material for: Term sets: A transparent and reproducible representation of clinical code sets
Source: PLoS One. 2019 Feb 14;14(2):e0212291. doi: 10.1371/journal.pone.0212291 (PMC6375602; doi:10.1371/journal.pone.0212291)
Supplement: S2 Appendix — Step by step construction of a term set for Type 2 Diabetes. (DOCX) [file pone.0212291.s003.docx]

Worked example of a Type 2 Diabetes term set

1. Inclusion terms: Type 2 diabetes / Exclusion terms: -

Matches 52 codes

C109012 Type 2 diabetes mellitus with renal complications

C109112 Type 2 diabetes mellitus with ophthalmic complications

C109212 Type 2 diabetes mellitus with neurological complications

C109312 Type 2 diabetes mellitus with multiple complications

…

71 unmatched descendants (codes in the hierarchy below matched terms, but that aren’t matched themselves)

C10F.11 Type II diabetes mellitus

C10FR11 Type II diabetes mellitus with gastroparesis

0 excluded codes.

Decision: Add “Type II diabetes” to the inclusion list

1. Inclusion terms: Type 2 diabetes, Type II diabetes / Exclusion terms: -

Matches 99 codes

24 unmatched descendants – most of which contain the phrase “non-insulin dependent diabetes mellitus”

0 excluded codes

Decision: Add “non-insulin dependent diabetes” to the inclusion list

1. Inclusion terms: Type 2 diabetes, Type II diabetes, non-insulin dependent diabetes / Exclusion terms: -

Matches 121 codes

6 unmatched descendants:

C10FS00 Maternally inherited diabetes mellitus

C10F800 Reaven's syndrome

C10F811 Metabolic syndrome X

C109800 Reaven's syndrome

C100100 Diabetes mellitus, adult onset, with no mention of complication

C100111 Maturity onset diabetes

0 excluded codes.

Decision: We’re not interested in maternally inherited diabetes, maturity onset diabetes, metabolic syndrome or reaven’s syndrome so add them to exclusion list. We do want adult onset though so add that to inclusion list.

1. Inclusion terms: Type 2 diabetes, Type II diabetes, non-insulin dependent diabetes, diabetes adult onset/ Exclusion terms: reaven's syndrome, metabolic syndrome, maternally inherited diabetes, maturity onset

Matches 130 codes of which the following are not indicative of Type 2 diabetes:

1252100 Family history of diabetes mellitus type 2

1252111 Family history of diabetes mellitus type II

66Ao.00 Diabetes type 2 review

C10P100 Type II diabetes mellitus in remission

C10P111 Type 2 diabetes mellitus in remission

L180B00 Pre-existing type 2 diabetes mellitus in pregnancy

0 unmatched descendants:

17 excluded codes

Decision: We add “family history”, “review”, “in remission” and “in pregnancy” to the exclusion list.

1. Inclusion terms: Type 2 diabetes, Type II diabetes, non-insulin dependent diabetes, diabetes adult onset/ Exclusion terms: reaven's syndrome, metabolic syndrome, maternally inherited diabetes, maturity onset

Matches 124 codes:

C100100 Diabetes mellitus, adult onset, with no mention of complication

C100112 Non-insulin dependent diabetes mellitus

C101100 Diabetes mellitus, adult onset, with ketoacidosis

C102100 Diabetes mellitus, adult onset, with hyperosmolar coma

C103100 Diabetes mellitus, adult onset, with ketoacidotic coma

C104100 Diabetes mellitus, adult onset, with renal manifestation

C105100 Diabetes mellitus, adult onset, with ophthalmic manifestation

C106100 Diabetes mellitus, adult onset, with neurological manifestation

C107100 Diabetes mellitus, adult onset, with peripheral circulatory disorder

C109.00 Non-insulin dependent diabetes mellitus

C109.11 NIDDM - Non-insulin dependent diabetes mellitus

C109.12 Type 2 diabetes mellitus

C109.13 Type II diabetes mellitus

C109000 Non-insulin-dependent diabetes mellitus with renal complications

C109011 Type II diabetes mellitus with renal complications

C109012 Type 2 diabetes mellitus with renal complications

C109100 Non-insulin-dependent diabetes mellitus with ophthalmic complications

C109111 Type II diabetes mellitus with ophthalmic complications

C109112 Type 2 diabetes mellitus with ophthalmic complications

C109200 Non-insulin-dependent diabetes mellitus with neurological complications

C109211 Type II diabetes mellitus with neurological complications

C109212 Type 2 diabetes mellitus with neurological complications

C109300 Non-insulin-dependent diabetes mellitus with multiple complications

C109311 Type II diabetes mellitus with multiple complications

C109312 Type 2 diabetes mellitus with multiple complications

C109400 Non-insulin dependent diabetes mellitus with ulcer

C109411 Type II diabetes mellitus with ulcer

C109412 Type 2 diabetes mellitus with ulcer

C109500 Non-insulin dependent diabetes mellitus with gangrene

C109511 Type II diabetes mellitus with gangrene

C109512 Type 2 diabetes mellitus with gangrene

C109600 Non-insulin-dependent diabetes mellitus with retinopathy

C109611 Type II diabetes mellitus with retinopathy

C109612 Type 2 diabetes mellitus with retinopathy

C109700 Non-insulin dependent diabetes mellitus - poor control

C109711 Type II diabetes mellitus - poor control

C109712 Type 2 diabetes mellitus - poor control

C109900 Non-insulin-dependent diabetes mellitus without complication

C109911 Type II diabetes mellitus without complication

C109912 Type 2 diabetes mellitus without complication

C109A00 Non-insulin dependent diabetes mellitus with mononeuropathy

C109A11 Type II diabetes mellitus with mononeuropathy

C109A12 Type 2 diabetes mellitus with mononeuropathy

C109B00 Non-insulin dependent diabetes mellitus with polyneuropathy

C109B11 Type II diabetes mellitus with polyneuropathy

C109B12 Type 2 diabetes mellitus with polyneuropathy

C109C00 Non-insulin dependent diabetes mellitus with nephropathy

C109C11 Type II diabetes mellitus with nephropathy

C109C12 Type 2 diabetes mellitus with nephropathy

C109D00 Non-insulin dependent diabetes mellitus with hypoglycaemic coma

C109D11 Type II diabetes mellitus with hypoglycaemic coma

C109D12 Type 2 diabetes mellitus with hypoglycaemic coma

C109E00 Non-insulin dependent diabetes mellitus with diabetic cataract

C109E11 Type II diabetes mellitus with diabetic cataract

C109E12 Type 2 diabetes mellitus with diabetic cataract

C109F00 Non-insulin-dependent diabetes mellitus with peripheral angiopathy

C109F11 Type II diabetes mellitus with peripheral angiopathy

C109F12 Type 2 diabetes mellitus with peripheral angiopathy

C109G00 Non-insulin dependent diabetes mellitus with arthropathy

C109G11 Type II diabetes mellitus with arthropathy

C109G12 Type 2 diabetes mellitus with arthropathy

C109H00 Non-insulin dependent diabetes mellitus with neuropathic arthropathy

C109H11 Type II diabetes mellitus with neuropathic arthropathy

C109H12 Type 2 diabetes mellitus with neuropathic arthropathy

C109J00 Insulin treated Type 2 diabetes mellitus

C109J11 Insulin treated non-insulin dependent diabetes mellitus

C109J12 Insulin treated Type II diabetes mellitus

C109K00 Hyperosmolar non-ketotic state in type 2 diabetes mellitus

C10D.00 Diabetes mellitus autosomal dominant type 2

C10F.00 Type 2 diabetes mellitus

C10F.11 Type II diabetes mellitus

C10F000 Type 2 diabetes mellitus with renal complications

C10F011 Type II diabetes mellitus with renal complications

C10F100 Type 2 diabetes mellitus with ophthalmic complications

C10F111 Type II diabetes mellitus with ophthalmic complications

C10F200 Type 2 diabetes mellitus with neurological complications

C10F211 Type II diabetes mellitus with neurological complications

C10F300 Type 2 diabetes mellitus with multiple complications

C10F311 Type II diabetes mellitus with multiple complications

C10F400 Type 2 diabetes mellitus with ulcer

C10F411 Type II diabetes mellitus with ulcer

C10F500 Type 2 diabetes mellitus with gangrene

C10F511 Type II diabetes mellitus with gangrene

C10F600 Type 2 diabetes mellitus with retinopathy

C10F611 Type II diabetes mellitus with retinopathy

C10F700 Type 2 diabetes mellitus - poor control

C10F711 Type II diabetes mellitus - poor control

C10F900 Type 2 diabetes mellitus without complication

C10F911 Type II diabetes mellitus without complication

C10FA00 Type 2 diabetes mellitus with mononeuropathy

C10FA11 Type II diabetes mellitus with mononeuropathy

C10FB00 Type 2 diabetes mellitus with polyneuropathy

C10FB11 Type II diabetes mellitus with polyneuropathy

C10FC00 Type 2 diabetes mellitus with nephropathy

C10FC11 Type II diabetes mellitus with nephropathy

C10FD00 Type 2 diabetes mellitus with hypoglycaemic coma

C10FD11 Type II diabetes mellitus with hypoglycaemic coma

C10FE00 Type 2 diabetes mellitus with diabetic cataract

C10FE11 Type II diabetes mellitus with diabetic cataract

C10FF00 Type 2 diabetes mellitus with peripheral angiopathy

C10FF11 Type II diabetes mellitus with peripheral angiopathy

C10FG00 Type 2 diabetes mellitus with arthropathy

C10FG11 Type II diabetes mellitus with arthropathy

C10FH00 Type 2 diabetes mellitus with neuropathic arthropathy

C10FH11 Type II diabetes mellitus with neuropathic arthropathy

C10FJ00 Insulin treated Type 2 diabetes mellitus

C10FJ11 Insulin treated Type II diabetes mellitus

C10FK00 Hyperosmolar non-ketotic state in type 2 diabetes mellitus

C10FK11 Hyperosmolar non-ketotic state in type II diabetes mellitus

C10FL00 Type 2 diabetes mellitus with persistent proteinuria

C10FL11 Type II diabetes mellitus with persistent proteinuria

C10FM00 Type 2 diabetes mellitus with persistent microalbuminuria

C10FM11 Type II diabetes mellitus with persistent microalbuminuria

C10FN00 Type 2 diabetes mellitus with ketoacidosis

C10FN11 Type II diabetes mellitus with ketoacidosis

C10FP00 Type 2 diabetes mellitus with ketoacidotic coma

C10FP11 Type II diabetes mellitus with ketoacidotic coma

C10FQ00 Type 2 diabetes mellitus with exudative maculopathy

C10FQ11 Type II diabetes mellitus with exudative maculopathy

C10FR00 Type 2 diabetes mellitus with gastroparesis

C10FR11 Type II diabetes mellitus with gastroparesis

C10y100 Diabetes mellitus, adult onset, with other specified manifestation

C10z100 Diabetes mellitus, adult onset, with unspecified complication

L180600 Pre-existing diabetes mellitus, non-insulin-dependent

0 unmatched descendants:

23 excluded codes:

C10D.11 Maturity onset diabetes in youth type 2

1252100 Family history of diabetes mellitus type 2

1252111 Family history of diabetes mellitus type II

C10P100 Type II diabetes mellitus in remission

C10P111 Type 2 diabetes mellitus in remission

L180B00 Pre-existing type 2 diabetes mellitus in pregnancy

66Ao.00 Diabetes type 2 review

C108900 Insulin dependent diabetes maturity onset

C10E912 Insulin dependent diabetes maturity onset

C10FS00 Maternally inherited diabetes mellitus

C10F800 Reaven's syndrome

C10F811 Metabolic syndrome X

C109800 Reaven's syndrome

C108911 Type I diabetes mellitus maturity onset

C108912 Type 1 diabetes mellitus maturity onset

C10E900 Type 1 diabetes mellitus maturity onset

C10E911 Type I diabetes mellitus maturity onset

C100111 Maturity onset diabetes

C108911 Type I diabetes mellitus maturity onset

C108912 Type 1 diabetes mellitus maturity onset

C10E900 Type 1 diabetes mellitus maturity onset

C10E911 Type I diabetes mellitus maturity onset

C100111 Maturity onset diabetes

Decision: We are finished.
